# Anxiety in patients with lower-extremity deep vein thrombosis: a patient-centred sequential explanatory mixed-methods study of prevalence, associated factors, and patient experiences

**DOI:** 10.3389/fmed.2026.1933822

**Published:** 2026-10-15

**Authors:** Yan Lin, Liqin Wang, Fengxiong Wang, Qiuni Cai

**Affiliations:** 1 Department of Rare Diseases, Zhongshan Hospital of Xiamen University, School of Medicine, Xiamen University, Xiamen, Fujian, China; 2 Department of Nephrology, Zhongshan Hospital of Xiamen University, School of Medicine, Xiamen University, Xiamen, Fujian, China; 3 Department of Foot and Ankle & Wound Healing, The First Affiliated Hospital of Guangdong Pharmaceutical University, Guangzhou, China; 4 Department of General Surgery, Zhongshan Hospital of Xiamen University, School of Medicine, Xiamen University, Xiamen, Fujian, China

**Keywords:** anxiety, deep vein thrombosis, GAD-7, illness perception, mixed-methods study, patient-reported outcomes, social support, venous thromboembolism

## Abstract

**Background:**

Lower extremity deep vein thrombosis (LEDVT) is associated with substantial psychological burden. Anxiety after DVT may reflect not only an emotional consequence of thrombosis, but also a signal of unmet informational, functional, and supportive care needs. This study aimed to investigate the prevalence and factors associated with anxiety among LEDVT patients and to explore their lived experiences, perceived triggers, and support needs using a mixed-methods design.

**Methods:**

A sequential explanatory mixed-methods study was conducted. In the quantitative phase, 175 LEDVT patients completed the Generalized Anxiety Disorder-7 (GAD-7), Brief Illness Perception Questionnaire (B-IPQ), and Multidimensional Scale of Perceived Social Support (MSPSS). Clinical and care-process variables were collected, including the perceived adequacy of structured counselling at diagnosis using an 8-item checklist. Clinically significant anxiety was defined as GAD-7 score ≥10. Multivariable logistic regression was performed according to a prespecified conceptual framework. In the qualitative phase, semi-structured interviews were conducted with 15 purposively selected participants; data were analyzed using thematic analysis.

**Results:**

The prevalence of clinically significant anxiety was 41.1% (72/175; 95% CI: 33.9%–48.6%). Younger age (OR = 0.95, 95% CI: 0.92–0.98, *P* = 0.002), higher B-IPQ score (OR = 1.18, 95% CI: 1.11–1.26, *P* < 0.001), lower MSPSS score (OR = 0.94, 95% CI: 0.90–0.98, *P* = 0.005), and adequate counselling at diagnosis (OR = 0.42, 95% CI: 0.20–0.88, *P* = 0.022) were independently associated with anxiety. Qualitative analysis generated five major themes: fear of complications, treatment-related anxiety, role and functional impairment, information delivery and illness meaning-making, and insufficient emotional and social support.

**Conclusion:**

Anxiety affects approximately two in five LEDVT patients in this single-centre cross-sectional cohort. Anxiety was associated not only with younger age, negative illness perception, and low social support, but also with symptom burden and perceived inadequacy of counselling at diagnosis; qualitative findings additionally highlighted symptom burden and functional limitations as key experiential drivers of anxiety. Integrated DVT care should address anticoagulation management, structured education, risk communication, cognitive reframing, functional recovery, and psychosocial support.

## 1 Introduction

Lower extremity deep vein thrombosis (LEDVT), the most common manifestation of venous thromboembolism (VTE), represents the third leading vascular disease globally, with an annual incidence of approximately 0.75–2.69 per 1,000 individuals (1, 2). While advanced age is a well-established risk factor, the incidence among young and middle-aged adults is rising due to increasing obesity, sedentary behavior, and occupational stress (3, 4). Beyond the immediate physical sequelae of limb swelling and pain, LEDVT imposes a significant psychological burden. Recent evidence indicates that approximately 50% of VTE patients experience clinically significant psychological distress, with anxiety rates threefold higher than the general population (5–8).

The relationship between VTE and anxiety is complex and potentially bidirectional. While earlier meta-analyses suggested anxiety may contribute to a hypercoagulable state (9), recent large-scale prospective data from the UK Biobank found no independent predictive effect of anxiety on incident VTE after rigorous confounder adjustment (10). This has shifted the clinical focus toward the psychological consequences of VTE diagnosis. Within this context, age emerges as a critical moderator. Longitudinal analyses by Steiner et al. (7) identified younger age as a associated factor of worsening anxiety trajectories following VTE. Furthermore, a preliminary study by Godoy et al. (8) reported a 40% prevalence of generalized anxiety disorder specifically in young VTE patients, underscoring the heightened psychological vulnerability of this demographic.

Importantly, anxiety after DVT should be interpreted within the broader framework of patient-centred and value-based VTE care. De Jong, Rosovsky, and Klok emphasized that the full picture of VTE outcomes includes not only conventional clinical endpoints such as death, recurrent VTE, and bleeding, but also patient-centred and societal outcomes, including quality of life, symptom burden, functional limitations, ability to work, psychosocial well-being, treatment satisfaction, and economic consequences (11). Similarly, the International Consortium for Health Outcomes Measurement VTE standard set and recent systematic reviews of VTE patient-reported outcomes highlight the need to routinely capture outcomes that matter to patients, including functional status, treatment burden, disease-specific quality of life, and mental health (12, 13). Core-outcome-set work in VTE treatment studies has also shown substantial heterogeneity in reported outcomes and supports broader incorporation of patient-important domains (14). Within this framework, anxiety is not an isolated psychological endpoint; rather, it may reflect the interaction among symptoms, perceived risk, information delivery, treatment burden, social context, and functional disruption.

Most existing research has relied exclusively on quantitative methods using standardized scales such as the Hospital Anxiety and Depression Scale (HADS) or Generalized Anxiety Disorder-7 (GAD-7). While these studies provide valuable prevalence estimates and statistical associations, they offer limited insight into patients’ lived experiences, perceived triggers, coping mechanisms, and unmet support needs. Moreover, few studies have explicitly examined whether anxiety is associated with the adequacy and framing of counselling at the time of DVT diagnosis. This is clinically important because fear of pulmonary embolism, recurrence, bleeding, post-thrombotic syndrome, and uncertain treatment duration may be amplified when information is incomplete, inconsistent, or communicated in a frightening manner.

Therefore, this study aimed to investigate the anxiety status and its associated factors among patients with LEDVT using a sequential explanatory mixed-methods design. The quantitative phase sought to determine the prevalence of anxiety and identify sociodemographic, clinical, care-process, and psychosocial factors associated with anxiety. The qualitative phase explored patients’ lived experiences of anxiety, perceived triggers, coping mechanisms, information needs, and support needs. By integrating both strands of inquiry, this study seeks to provide a more nuanced understanding of anxiety after DVT and to inform holistic, patient-centred follow-up care.

## 2 Methods

A sequential explanatory mixed-methods design was employed, comprising two distinct phases (15). Phase I consisted of a cross-sectional quantitative survey to determine the prevalence of anxiety and identify associated demographic and clinical factors. Phase II involved semi-structured individual interviews to explore in depth the subjective experiences and perceived determinants of anxiety identified in the quantitative phase. The explanatory design was chosen so that the qualitative findings could help interpret and contextualize the unexpected or incomplete patterns observed in the quantitative survey data (16). The quantitative component is reported in accordance with the Strengthening the Reporting of Observational Studies in Epidemiology (STROBE) statement (17), and the qualitative component in accordance with the Consolidated Criteria for Reporting Qualitative Research (COREQ) (18).

### 2.1 Study setting and participants

This study was conducted at the Department of Vascular Surgery, Zhongshan Hospital, Xiamen University, a general hospital, between August 2024 and May 2025. Eligible participants were identified through the hospital's electronic medical record system and outpatient clinic schedules.

Inclusion criteria for both phases were: ① age ≥18 years; ② a confirmed diagnosis of acute or subacute lower extremity deep vein thrombosis (DVT) by compression ultrasonography or computed tomography venography within the preceding 12 months; ③ current prescription of anticoagulant therapy; and ④ the ability to provide written informed consent and communicate in Mandarin Chinese.

Exclusion criteria were: ① a documented history of severe psychiatric illness (e.g., schizophrenia, bipolar disorder) or current substance abuse that would impede participation; ② Coexisting severe functional failure of vital organs such as the heart, brain, liver, or kidneys, or being in a hemodynamically unstable state; ③ Women who are pregnant or in the puerperium; and ④ Participation in other clinical trials involving psychological interventions.

### 2.2 Phase I: quantitative component

#### 2.2.1 Sampling and data collection

For the cross-sectional survey, consecutive patients attending the vascular surgery outpatient clinic during the study period who met eligibility criteria were invited to participate. After obtaining informed consent, participants completed a structured questionnaire battery either in a private consultation room or online via a secure survey platform.

#### 2.2.2 Variables and instruments

The following data were collected:

(1) Demographic and clinical characteristics: A standardized case report form was used to collect age, sex, body mass index, educational level, employment status, monthly household income, marital status, having children, and place of residence. Clinical variables extracted from medical records included DVT laterality, DVT extent, concomitant pulmonary embolism at diagnosis, time since diagnosis, anticoagulant type, previous VTE.
(2) Structured counselling at diagnosis: To address the potential confounding effect of heterogeneous information delivery, counselling at DVT diagnosis was assessed using an 8-item Structured Counselling Checklist. The checklist recorded whether patients recalled receiving explanations regarding: ① the nature and causes of DVT; ② pulmonary embolism risk and warning signs; ③ recurrence risk and prevention; ④ bleeding risk and management; ⑤ anticoagulation duration and follow-up plan; ⑥ post-thrombotic syndrome and expected symptom trajectory; ⑦ written or electronic educational materials; and ⑧ opportunity to ask questions with confirmation of understanding. Each item was scored 0 or 1, yielding a total score of 0–8. Adequate counselling was defined *a priori* as a score ≥6. The complete checklist is provided as Supplementary Materials S1.
(3) Anxiety Assessment: The primary outcome, anxiety symptoms over the past two weeks, was measured using the Generalized Anxiety Disorder-7 (GAD-7) scale. The GAD-7 is a well-validated 7-item self-report instrument with scores ranging from 0 to 21; a cut-off score of ≥10 was used to define clinically significant anxiety symptoms, demonstrating a sensitivity of 89% and specificity of 82% in primary care populations (19). A GAD-7 score ≥10 indicates clinically significant anxiety symptoms; it does not by itself establish a formal diagnosis of an anxiety disorder. The Chinese version of the GAD-7 has demonstrated robust internal consistency (Cronbach's *α* = 0.90) and construct validity (20).
(4) Illness Perception: The Brief Illness Perception Questionnaire (B-IPQ)was used to evaluate patients’ cognitive and emotional representations of their DVT, including perceived consequences, timeline, personal control, treatment control, symptom identity, concern, understanding, and emotional response (21). A Chinese version/adaptation has shown acceptable psychometric performance in Chinese patients (22). Higher total scores indicate more threatening or negative illness perception.
(5) Social Support: The Multidimensional Scale of Perceived Social Support (MSPSS) assessed support from family, friends, and significant others (23). Higher scores indicate greater perceived social support.

#### 2.2.3 Statistical analysis

Descriptive statistics were performed for categorical variables using frequencies (percentages), and for continuous variables using means (standard deviations). The detection rate of anxiety symptoms (defined as a score ≥10 on the 7-item Generalized Anxiety Disorder scale) was calculated with a 95% confidence interval. Depending on the data characteristics, bivariate associations between anxiety status and demographic and clinical variables were analyzed using independent samples t-test, Mann-Whitney U test, chi-square test, or Fisher's exact test. Multivariable logistic regression was developed according to a prespecified conceptual framework rather than relying solely on univariate statistical significance. Candidate associated factors were grouped into four domains: demographic vulnerability, information and counselling exposure, and psychosocial resources. Given 72 anxiety events, the primary model was restricted to clinically justified covariates to reduce overfitting: age, concomitant pulmonary embolism, unemployment/sick leave/retirement, time since diagnosis, adequacy of counselling, B-IPQ total score, and MSPSS total score. Multicollinearity was assessed using variance inflation factors. For all final models, a two-sided *P* value <0.05 was considered statistically significant. Quantitative data were analyzed using SPSS 28.0.

#### 2.2.4 Sample size justification

Based on a conservative estimate of 35% prevalence of clinically significant anxiety symptoms in the DVT population derived from prior literature (24), a minimum sample size of 156 participants was required (*α* = 0.05), corresponding to an absolute margin of error of approximately ±7.5% for the prevalence estimate. Anticipating a 15% rate of incomplete questionnaires, the target recruitment was set at 180 participants; the achieved final sample of 175 exceeded the minimum requirement.

### 2.3 Phase II: qualitative component

#### 2.3.1 Sampling and recruitment

Participants for the qualitative phase were purposively selected from the quantitative cohort using a maximum-variation sampling matrix. The matrix was developed *a priori* to capture heterogeneity in dimensions considered clinically and psychosocially relevant to the experience of DVT, including age group, sex, GAD-7 anxiety severity, DVT extent, presence of concomitant pulmonary embolism, anticoagulant type, time since diagnosis, employment status, and perceived adequacy of counselling at diagnosis. Among 94 survey participants who consented to be contacted for interviews, 22 were approached and 15 agreed to participate (68.2%). The main reasons for non-participation among the remaining seven patients were time constraints due to work or family commitments (*n* = 3), reluctance to discuss personal emotional experiences (*n* = 2), and perceived lack of need (*n* = 2). Recruitment continued until meaning saturation was achieved, operationally defined as the point at which no new codes or substantially new interpretations relevant to the study aims emerged in three consecutive interviews. Three additional interviews were conducted after preliminary saturation to confirm informational redundancy. The maximum-variation sampling matrix is provided as Supplementary Materials S2 and the saturation grid as Supplementary Materials S3.

#### 2.3.2 Data collection

Individual semi-structured interviews were conducted by a trained qualitative researcher who had no clinical relationship with the participants. The interview guide was developed based on the quantitative findings and existing VTE patient-outcome literature and was refined through pilot interviews with two patients with DVT. The guide explored: 1) personal experiences of being diagnosed with and living with DVT; 2) perceptions of pulmonary embolism, recurrence, bleeding, post-thrombotic syndrome, and prognosis; 3) emotional responses to anticoagulation management; 4) impact on daily activities, family roles, work, and finances; 5) information received at diagnosis and perceptions of clinician communication; 6) sources of social and professional support; and 7) unmet supportive care needs. Interviews were conducted in a hospital consultation room, by telephone, or by online video platform according to participant preference, lasted 30–50 min, and were audio-recorded with explicit consent. Field notes were written immediately after each interview. The full interview guide is provided as Supplementary Materials S4.

#### 2.3.3 Qualitative data analysis

Interviews were transcribed verbatim in Mandarin Chinese and checked for accuracy against audio recordings by the interviewer. Data were managed and analyzed using NVivo 14. Thematic analysis was conducted following the six-phase framework proposed by Braun and Clarke (25). Two researchers independently read and coded the first five transcripts to develop a preliminary coding framework, which was discussed and refined in a consensus meeting. The coding framework was then applied to all remaining transcripts, with regular team meetings to review coding decisions and resolve discrepancies.

Coding disagreements were resolved through discussion and consensus, with reference to the original transcripts; where consensus could not be reached, the disputed code was discussed with a third member of the research team. Codes were organized into descriptive subthemes and overarching analytical themes.

Several strategies were used to enhance trustworthiness. First, maximum-variation sampling was used to improve transferability. Second, two researchers independently coded initial transcripts, and discrepancies were resolved by discussion. Third, an audit trail documented coding decisions, theme development, and changes to the interview guide. Fourth, reflexive memos were maintained throughout data collection and analysis. Fifth, peer debriefing was conducted with clinicians and qualitative researchers not directly involved in coding. Finally, respondent validation was performed by returning a concise summary of preliminary themes to 15 interview participants; 12 responded, 10 confirmed the themes, and 2 provided minor clarifications that were incorporated into the final interpretation.

Translation of quotations. Interviews were transcribed, coded, and analyzed in Mandarin Chinese. The participant quotations presented in this article were translated from Mandarin into English by YL and LQW, and translation accuracy was checked through consensus discussion between translators. Reflexivity. The interviewer was a trained qualitative researcher with no clinical role in participants’ care. Reflexive memos documented how the interviewer's professional background, assumptions, and relationship with the topic might influence data collection and interpretation, and these reflections were reviewed in team meetings to limit their influence on coding and theme development.

### 2.4 Integration and mixed-methods analysis

Integration of quantitative and qualitative findings occurred at three stages (Figure 1). First, quantitative results informed purposive sampling and refinement of the qualitative interview guide.

**Figure 1:**
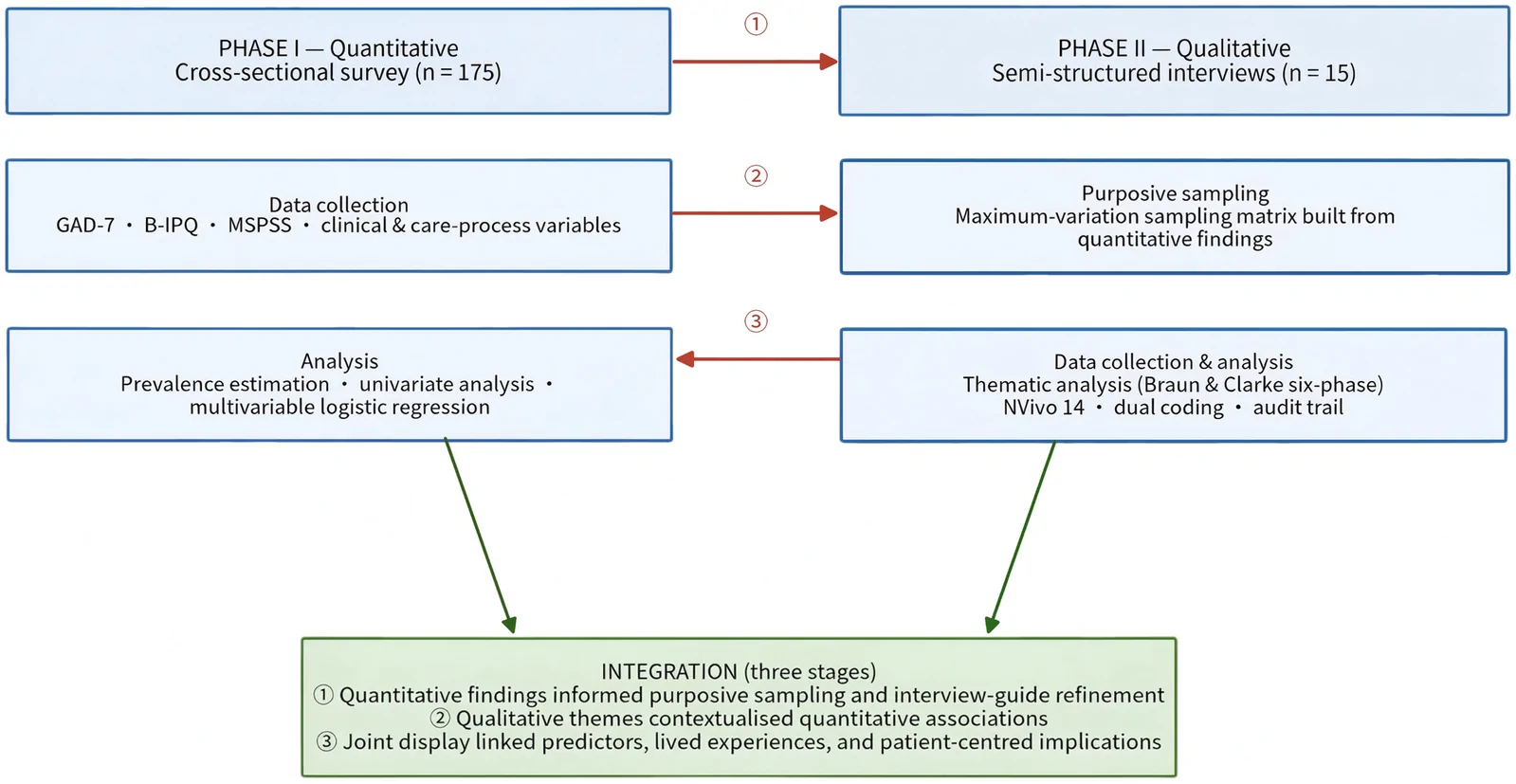
Sequential explanatory mixed-methods design. GAD-7, Generalized Anxiety Disorder-7; B-IPQ, Brief illness Perception Questionnaire; MSPSS, Multidimensional Scale of Perceived Social Support.

Specifically, because the quantitative phase identified younger age and non-employment as associated factors, the guide included detailed probes on the impact of DVT on work, finances, and family roles (guide domain 4); because perceived inadequacy of counselling at diagnosis was associated with anxiety, the guide included detailed probes on information received at diagnosis and perceptions of clinician communication (guide domain 5); and because lower perceived social support was associated with anxiety, the guide explored sources of social and professional support (guide domain 6). Second, qualitative themes were used to explain and contextualize quantitative associations. Third, a joint display was constructed to integrate statistical associations, qualitative themes, and patient-centred VTE outcome domains.

### 2.5 Ethical considerations

The study protocol was approved by the Institutional Review Board of Zhongshan Hospital, Xiamen University (Approval No.: 2022-085) and conducted in accordance with the Declaration of Helsinki. All participants provided written informed consent after receiving a full explanation of the study purposes, procedures, voluntary nature of participation, and confidentiality measures. To protect privacy, all data were de-identified and stored on password-protected, encrypted institutional servers accessible only to the research team.

## 3 Results

### 3.1 Participant characteristics (quantitative phase)

A total of 210 consecutive patients with LEDVT were screened for eligibility between August 2024 and May 2025. Of these, 195 met the inclusion criteria and were invited to participate. Twelve patients declined participation, and 8 submitted incomplete questionnaires, resulting in a final sample of 175 participants (effective response rate: 89.7%).

The demographic and clinical characteristics of the participants are summarized in Table 1. The mean age was 54.6 years (SD = 14.2, range 21–78), with a slight preponderance of male patients (*n* = 96, 54.9%). The majority were married (*n* = 142, 81.1%) and had an educational level of high school or above (*n* = 103, 58.9%). Regarding clinical features, the median time since DVT diagnosis was 3.5 months (IQR 1.5–7.0). Left-sided DVT was most common (*n* = 98, 56.0%), and 28 patients (16.0%) had concomitant PE at diagnosis. Most patients were prescribed direct oral anticoagulants (DOACs) (*n* = 132, 75.4%).

**Table 1:** Demographic and clinical characteristics of participants (*N* = 175).

Variable	Value [mean ± SD/n (%)/median (IQR)]
Age (years)	54.6 ± 14.2
Gender	
Male	96 (54.9)
Female	79 (45.1)
BMI (kg/m^2^)	25.8 ± 4.1
Education level	
Junior high school or below	72 (41.1)
High school or above	103 (58.9)
Employment status	
Employed	98 (56.0)
Unemployed/Retired/Sick leave	77 (44.0)
Monthly household income (CNY)	
<5,000	45 (25.7)
5,000–10,000	78 (44.6)
>10,000	52 (29.7)
Marital status	
Married	142 (81.1)
Single/Divorced/Widowed	33 (18.9)
Having children	
Yes	138 (78.9)
No	37 (21.1)
Residence	
Urban	121 (69.1)
Rural	54 (30.9)
DVT laterality	
Left	98 (56.0)
Right	58 (33.1)
Bilateral	19 (10.9)
DVT location	
Proximal	113 (64.6)
Distal	62 (35.4)
Adequate counselling	
Yes	98 (56.0)
No	77 (44.0)
Previous VTE	
Yes	22 (12.6)
No	153 (87.4)
Concomitant PE at diagnosis	28 (16.0)
Time since diagnosis (months)	3.5 (1.5–7.0)
Anticoagulant type	
DOACs	132 (75.4)
VKAs	43 (24.6)
GAD-7 score	8.4 ± 5.3
B-IPQ total score	41.8 ± 11.6
MSPSS total score	56.2 ± 13.4

BMI, body mass index; CNY, Chinese Yuan; DVT, deep vein thrombosis; PE, pulmonary embolism; IQR, interquartile range; DOACs, direct oral anticoagulants; VKAs, vitamin K antagonists; GAD-7, Generalized Anxiety Disorder-7 scale; B-IPQ, Brief Illness Perception Questionnaire; MSPSS, Multidimensional Scale of Perceived Social Support.

### 3.2 Prevalence of anxiety and univariate analysis

The mean GAD-7 score for the participants was 8.4 (SD = 5.3). Using the established cut-off score of ≥10, 72 participants (41.1%; 95% CI: 33.9%–48.6%) were identified as having clinically significant anxiety symptoms. In terms of GAD-7 severity categories, 38 (21.7%) participants scored within the minimal range (0–4), 65 (37.1%) within the mild range (5–9), 49 (28.0%) within the moderate range (10–14), and 23 (13.1%) within the severe range (15–21).

Univariate analysis revealed significant associations between anxiety and several variables (Table 2). Participants with anxiety were significantly younger and more likely to be unemployed or on sick leave. The presence of concomitant PE at diagnosis was also associated with a higher proportion of anxiety. Regarding psychosocial measures, anxious patients reported significantly higher B-IPQ total scores and lower MSPSS total scores. No significant differences were observed between the groups regarding gender, thrombus location, or duration of anticoagulation.

**Table 2:** Univariate analysis of factors associated with anxiety symptoms (GAD-7 ≥ 10) (*N* = 175).

Variable	Category	Non-anxiety (*n* = 103)	Anxiety (*n* = 72)	*P*-value
Age (years)	-	58.4 ± 12.9	49.2 ± 14.1	<0.001
Gender	Male	55 (53.4%)	41 (56.9%)	0.542
	Female	48 (46.6%)	31 (43.1%)	
BMI (kg/m^2^)	-	25.6 ± 4.0	26.1 ± 4.3	0.431
Education level	Junior high school or below	40 (38.8%)	32 (44.4%)	0.457
	High school or above	63 (61.2%)	40 (55.6%)	
Employment status	Employed	66 (64.1)	32 (44.4)	0.009
	Unemployed/Sick leave/Retired	37 (35.9)	40 (55.6)	
Monthly household income (CNY)	<5,000	22 (21.4%)	23 (31.9%)	0.114
	5,000–10,000	50 (48.5%)	28 (38.9%)	
	>10,000	31 (30.1%)	21 (29.2%)	
Marital status	Married	88 (85.4%)	54 (75.0%)	0.082
	Single/Divorced/Widowed	15 (14.6%)	18 (25.0%)	
Having children	Yes	84 (81.6%)	54 (75.0%)	0.294
	No	19 (18.4%)	18 (25.0%)	
Residence	Urban	72 (69.9%)	49 (68.1%)	0.796
	Rural	31 (30.1%)	23 (31.9%)	
DVT laterality	Left	60 (58.3%)	38 (52.8%)	0.673
	Right	32 (31.1%)	26 (36.1%)	
	Bilateral	11 (10.7%)	8 (11.1%)	
DVT location	Proximal	63 (61.2%)	50 (69.4%)	0.256
	Distal	40 (38.8%)	22 (30.6%)	
Previous VTE	Yes	9 (8.7)	13 (18.1)	0.068
	No	94 (91.3)	59 (81.9)	
Concomitant PE at diagnosis	Yes	11 (10.7%)	17 (23.6%)	0.022
	No	92 (89.3%)	55 (76.4%)	
Time since diagnosis (months)	-	3.8 (1.8–7.5)	3.2 (1.2–6.5)	0.185
Anticoagulant type	DOACs	78 (75.7%)	54 (75.0%)	0.912
	VKAs	25 (24.3%)	18 (25.0%)	
Adequate counselling	Yes	70 (68.0)	28 (38.9)	<0.001
	No	33 (32.0)	44 (61.1)	
B-IPQ total score	-	35.6 ± 8.9	48.2 ± 10.2	<0.001
MSPSS total score	-	61.8 ± 11.2	48.5 ± 12.6	<0.001

BMI, body mass index; CNY, Chinese Yuan; DVT, deep vein thrombosis; PE, pulmonary embolism; DOACs, direct oral anticoagulants; VKAs, vitamin K antagonists; B-IPQ, Brief Illness Perception Questionnaire; MSPSS, Multidimensional Scale of Perceived Social Support.

### 3.3 Factors associated with anxiety (multivariate analysis)

Variables associated with anxiety at *P* < 0.05 in univariate analysis (younger age, unemployment/sick leave/retirement, concomitant PE at diagnosis, perceived inadequacy of counselling, higher B-IPQ total score, and lower MSPSS total score), together with time since diagnosis (retained *a priori* as a clinically important covariate), were entered into a multivariable logistic regression model. The results are presented in Table 3. Younger age (OR = 0.95, 95% CI: 0.92–0.98, *P* = 0.002), higher B-IPQ score (OR = 1.18, 95% CI: 1.11–1.26, *P* < 0.001), adequate counselling at diagnosis (OR = 0.42, 95% CI: 0.20–0.88, *P* = 0.022) and lower perceived social support (MSPSS score; OR = 0.94, 95% CI: 0.90–0.98, *P* = 0.005) remained independently associated with clinically significant anxiety; employment status (OR = 1.68, 95% CI: 0.82–3.45, *P* = 0.158), concomitant PE (OR = 1.95, 95% CI: 0.91–4.18, *P* = 0.087), and time since diagnosis (OR = 0.98, 95% CI: 0.91–1.06, *P* = 0.626) were not statistically significant in the multivariable model. Expressed per one-standard-deviation increase, the OR for B-IPQ (SD = 11.6) was 6.82 (95% CI: 3.36–14.60), and the OR for MSPSS (SD = 13.4) was 0.44 (95% CI: 0.24–0.76). No automated stepwise variable-selection procedure was used.

**Table 3:** Multivariate logistic regression analysis of factors independently associated with clinically significant anxiety (GAD-7 ≥ 10).

Variable	B	SE	Wald	OR (95% CI)	*P*-value
Constant	−2.845	2.014	2.00	0.058	0.158
Age (years)	−0.051	0.017	9.18	0.95 (0.92–0.98)	0.002
Unemployed/Sick leave/Retired	0.519	0.367	2.00	1.68 (0.82–3.45)	0.158
Concomitant PE	0.667	0.389	2.94	1.95 (0.91–4.18)	0.087
Time since diagnosis	−0.018	0.037	0.24	0.98 (0.91–1.06)	0.626
Adequate counselling at diagnosis	−0.867	0.379	5.23	0.42 (0.20–0.88)	0.022
B-IPQ total score	0.165	0.032	26.58	1.18 (1.11–1.26)	< 0.001
MSPSS total score	−0.062	0.022	7.94	0.94 (0.90–0.98)	0.005

Hosmer–Lemeshow *χ*^2^ = 6.47, df = 8, *P* = 0.594; Nagelkerke R^2^ = 0.486; B, unstandardized regression coefficient (log-odds); maximum VIF = 2.1; SE, standard error; Wald, Wald statistic; OR, odds ratio; CI, confidence interval; PE, pulmonary embolism; B-IPQ, Brief Illness Perception Questionnaire; MSPSS, Multidimensional Scale of Perceived Social Support.

### 3.4 Qualitative findings

A purposive sample of 15 participants from the quantitative phase was recruited for semi-structured interviews. Thematic saturation was reached after the 12th interview; three additional interviews were conducted to confirm that no new codes or themes emerged. The sample comprised 8 women and 7 men, with ages ranging from 24 to 70 years. Detailed participant characteristics are presented in Table 4.

**Table 4:** Characteristics of qualitative interview participants (*N* = 15).

ID	Gender	Age (years)	DVT location	PE	Anticoagulant	Time since diagnosis (months)	Employment	Counselling adequacy
P01	Male	35	Proximal	No	DOAC	2	Employed	Inadequate
P02	Female	68	Distal	No	DOAC	5	Retired	Adequate
P03	Female	52	Proximal	Yes	DOAC	1	Sick leave	Inadequate
P04	Male	70	Proximal	No	VKA	6	Retired	Inadequate
P05	Male	28	Distal	No	DOAC	2	Employed	Adequate
P06	Female	31	Proximal	No	DOAC	1.2	Sick leave	Inadequate
P07	Female	65	Distal	No	VKA	8	Retired	Inadequate
P08	Male	47	Proximal	No	DOAC	3	Employed	Inadequate
P09	Male	45	Proximal	No	DOAC	4	Sick leave	Inadequate
P10	Female	39	Distal	No	DOAC	7	Employed	Adequate
P11	Male	42	Proximal	No	DOAC	2.5	Employed	Inadequate
P12	Female	24	Proximal	No	VKA	1	Employed	Inadequate
P13	Male	56	Distal	No	DOAC	10	Employed	Adequate
P14	Female	38	Proximal	No	DOAC	3	Employed	Adequate
P15	Female	60	Distal	No	DOAC	9	Retired	Adequate

Thematic analysis yielded 5 overarching themes and 14 subthemes (Table 5). Representative quotations are provided below.

**Table 5:** Qualitative themes and subthemes.

Overarching theme	Subthemes
1. Fear of complications	1.1 Fear of pulmonary embolism
	1.2 Worry about post-thrombotic syndrome
	1.3 Fear of recurrence
2. Treatment-Related Anxiety	2.1 Distress over bleeding risks
	2.2 Uncertainty about treatment duration
	2.3 Treatment burden and lifestyle restrictions
3. Role and functional impairment	3.1 Loss of independence and family role
	3.2 Financial pressure
	3.3 Functional limitations and symptom vigilance
4. Information delivery and illness meaning-making	4.1 Lack of structured disease education
	4.2 Frightening or fragmented risk communication
	4.3 Need for individualized explanations and written materials
5. Insufficient emotional and social support	5.1 Perceived insufficient emotional support from clinicians
	5.2 Family and peer support as coping resources

#### 3.4.1 Theme 1: fear of complications

Patients reported persistent anxiety driven by perceived life-threatening risks, with three subthemes.

##### 3.4.1.1 Sub-theme 1.1: Fear of pulmonary embolism

Participants described overwhelming worry about thrombus dislodgement leading to fatal PE, triggered by limb swelling, pain, or restricted movement.

“The doctor said the clot could move to my lung and kill me at any time. I dare not turn over or walk.” (P07)

##### 3.4.1.2 Sub-theme 1.2: Worry about post-thrombotic syndrome

Anxiety centered on long-term disability, and chronic pain, threatening daily function and quality of life.

“I’m scared my leg will stay swollen forever, turn black, and I won't be able to work or take care of my family.” (P11)

##### 3.4.1.3 Sub-theme 1.3: Fear of recurrence

Some participants perceived DVT as a disease that could recur unpredictably, particularly when the cause of thrombosis was not clearly explained.

“Even if this clot disappears, I keep wondering whether another one is already forming. I do not know what I did wrong.” (P05)

#### 3.4.2 Theme 2: treatment-related anxiety

Anxiety was closely tied to anticoagulant therapy, and unclear prognosis, forming three subthemes.

##### 3.4.2.1 Sub-theme 2.1: Distress over bleeding risks

Fears of spontaneous bleeding, and frequent monitoring caused persistent vigilance and distress.

“I take warfarin and check my blood every week. I’m afraid to bump into anything; even a small cut makes me panic.” (P04)

##### 3.4.2.2 Sub-theme 2.2: Uncertainty about treatment duration and recurrence

Lack of clarity on recovery timeline and high recurrence risk led to chronic anticipatory anxiety.

“No one tells me when I can stop medicine. I keep thinking: will it come back? ” (P09)

##### 3.4.2.3 Subtheme 2.3: Treatment burden and lifestyle restrictions

Participants reported avoiding exercise, social activities, alcohol, travel, or physical work because they were unsure what was safe during anticoagulation.

“I used to walk after dinner, but now I am afraid that walking too much will make the clot move. I also fear falling while taking anticoagulants.” (P14)

#### 3.4.3 Theme 3: role and functional impairment

Anxiety was amplified by disrupted social roles, economic burden, and insufficient support, with three subthemes.

##### 3.4.3.1 Sub-theme 3.1 Loss of independence and family role

Inability to work, care for family, or perform daily activities eroded self-efficacy and worsened emotional distress.

“I used to do everything for my family. Now I’m a burden; I feel useless and anxious all day.” (P02)

##### 3.4.3.2 Sub-theme 3.2 financial pressure

Medical costs, lost income, and limited family or community support exacerbated anxiety and hopelessness.

“Treatment is expensive, and I can't work. My family is stressed.” (P08)

##### 3.4.3.3 Subtheme 3.3: Functional limitations and symptom vigilance

Persistent leg symptoms made patients monitor their body closely, which reinforced anxiety.

“Every time my leg becomes swollen, I start checking whether it is another clot. I cannot focus on anything else.” (P03)

#### 3.4.4 Theme 4: information delivery and illness meaning-making

This theme was developed to address how counselling adequacy and risk communication shaped patients’ anxiety.

##### 3.4.4.1 Subtheme 4.1: Lack of structured disease education

Confusion about disease etiology, treatment trajectory, warning signs, and safe activity fueled uncertainty and rumination.

“I searched online but got scary information. The doctor explained too fast; I still don't understand what's happening to my body.” (P05)

##### 3.4.4.2 Subtheme 4.2: Frightening or fragmented risk communication

Some participants remembered risk information as abrupt or catastrophic, which intensified fear of sudden death even after clinical stabilization.

“I only remembered the word ‘fatal’. After that, every small discomfort made me think I might die.” (P12)

##### 3.4.4.3 Subtheme 4.3: Need for individualized explanations and written materials

Participants wanted clear, repeated, and individualized explanations regarding PE warning signs, recurrence prevention, bleeding management, treatment duration, and expected recovery.

“If I had a written list telling me what is dangerous and what is normal, I would not need to search online every night.” (P10)

#### 3.4.5 Theme 5: insufficient emotional and social support

Poor emotional support and fragmented provider communication heightened anxiety.

##### 3.4.5.1 Sub-theme 5.1: Perceived insufficient emotional support from clinicians

Participants desired reassurance and empathic communication, not only biomedical information.

“I need someone to comfort me, not just list risks.” (P03)

##### 3.4.5.2 Sub-theme 5.2: Family and peer support as coping resources

Participants with lower anxiety often described practical and emotional support from family members or other patients as a source of reassurance.

“My family reminds me to take medicine and go to follow-up. When someone explains things with me, I feel less afraid.” (P13)

### 3.5 Integration of quantitative and qualitative findings

The mixed-methods integration demonstrates a coherent relationship between quantitative associated factors and qualitative themes (Table 6). Younger age was quantitatively associated with anxiety, and qualitative interviews suggested that younger patients experienced greater distress related to employment interruption, financial pressure, and disrupted family roles. Higher B-IPQ scores were associated with anxiety, and qualitative themes showed that threatening illness representations were shaped by fear of PE, recurrence, PTS, and uncertainty about prognosis. Lower MSPSS scores were associated with anxiety, and qualitative findings revealed the importance of family support, peer reassurance, and clinician empathy.

**Table 6:** Joint display integrating quantitative and qualitative findings.

Quantitative finding	Qualitative explanation	Patient-centred VTE outcome domain	Clinical implication
Younger age associated with anxiety	Younger participants described work interruption, financial pressure, parenting burden, and identity disruption	Ability to work; societal outcomes; role functioning	Provide return-to-work guidance and age-tailored counselling
Higher B-IPQ score associated with anxiety	Patients held catastrophic beliefs about PE, recurrence, bleeding, and PTS	Psychosocial well-being; illness understanding	Use cognitive reframing and structured risk communication
Lower MSPSS score associated with anxiety	Family support and clinician reassurance buffered fear	Social support; treatment adherence	Involve family and improve clinician empathy
Inadequate counselling associated with anxiety	Fragmented or frightening explanations intensified perceived risk	Treatment satisfaction; health literacy; patient experience	Implement structured counselling checklist and teach-back

## 4 Discussion

This sequential explanatory mixed-methods study examined the prevalence, associated factors, and lived experience of anxiety among patients with LEDVT. The quantitative phase showed that 41.1% of participants had clinically significant anxiety symptoms. In the quantitative analysis, anxiety was independently associated with younger age, more threatening illness perception, lower perceived social support, and inadequate counselling at diagnosis. The qualitative phase provided explanatory depth, identifying fear of complications, treatment-related anxiety, role and functional impairment, information delivery and illness meaning-making, and insufficient support as key experiential domains.

The quantitative phase revealed that 41.1% of participants experienced clinically significant anxiety symptoms (GAD-7 ≥ 10), a figure that aligns closely with previous reports of psychological distress in VTE populations. For instance, Hunter et al. documented that approximately 50% of VTE patients report substantial psychological sequelae (5), while a recent study by Steiner et al. found that younger VTE patients exhibited worsening anxiety trajectories over time (7).

Younger age was strongly associated with anxiety. The qualitative narratives shed light on this association: younger participants frequently described profound distress over disrupted career trajectories, financial strain, and the sudden transition from being a family provider to feeling like a “burden.” This echoes previous research identifying younger age as a associated factor of worsening anxiety following VTE (26). From a developmental perspective, younger adults typically possess fewer coping resources and face more role demands, making a sudden chronic illness particularly disruptive to identity and future planning (27).

Negative illness perception showed the strongest association with anxiety. This finding supports Leventhal's Common-Sense Model of Self-Regulation, in which threatening cognitions and emotional representations of illness give rise to emotional distress (28). Qualitative data revealed that inadequate understanding of disease mechanisms and prognosis led to catastrophic thinking and rumination, a pattern also documented in qualitative studies of “post-thrombotic panic syndrome” (5). These insights suggest that illness perceptions may be a potentially modifiable target for intervention in LEDVT follow-up; however, because the quantitative data are cross-sectional, the observed association cannot be interpreted as evidence of a causal mechanism, and this interpretation is therefore framed as hypothesis-generating. Dimension-level analyses of the B-IPQ were not performed in this study; whether particular domains (e.g., perceived consequences, concern, or emotional response) contribute disproportionately to the observed association warrants investigation in future research.

The protective effect of higher perceived social support against anxiety was another important quantitative finding, resonating with the qualitative narratives. Participants with low anxiety scores frequently cited practical and emotional support from family members as a key coping resource. However, the qualitative data also revealed a dimension of support not fully captured by the MSPSS: the need for empathetic communication from healthcare providers. The subtheme of “insufficient perceived emotional support” highlighted patients’ desire for reassurance and compassion from clinicians. This finding suggests that while the MSPSS effectively measures support from family and friends, the therapeutic alliance with the care team constitutes a distinct and clinically significant source of support.

A key addition of the revised analysis is the explicit assessment of counselling adequacy at diagnosis. In the final multivariate model, adequate counselling was associated with substantially lower odds of anxiety even after adjustment for clinical severity and psychosocial factors. This finding directly addresses the concern that anxiety after DVT may partly reflect heterogeneous information delivery rather than intrinsic psychosocial consequences of thrombosis. The qualitative findings support this interpretation: some patients remembered receiving fragmented or frightening risk information, while others reported searching online because they did not understand recurrence risk, bleeding risk, PTS, treatment duration, or safe activities.

### 4.1 Integrated interpretation of quantitative and qualitative findings

The joint display (Table 6) links each quantitative association to its corresponding qualitative explanation and patient-centred implication. First, the association between younger age and anxiety was explained by qualitative accounts of employment interruption, financial pressure, parenting burden, and identity disruption; this maps to the patient-centred domains of ability to work and societal outcomes, and supports age-tailored return-to-work guidance and counselling. Second, the association between more threatening illness perception (higher B-IPQ) and anxiety was explained by catastrophic beliefs about PE, recurrence, bleeding, and PTS, which were reinforced by fragmented or frightening information delivery; this maps to psychosocial well-being and illness understanding, and supports cognitive reframing and structured risk communication. Third, the association between lower perceived social support and anxiety was explained by the protective role of family support and the unmet need for clinician reassurance; this maps to social support and treatment adherence, and supports the active involvement of family members and improved clinician empathy. Fourth, the association between perceived inadequacy of counselling at diagnosis and anxiety was explained by fragmented or frightening explanations that intensified perceived risk; this maps to treatment satisfaction, health literacy, and patient experience, and supports the implementation of a structured counselling checklist with teach-back. Together, these integrated findings indicate that anxiety after LEDVT is a multi-faceted experience shaped by the interaction of personal vulnerability, illness beliefs, information delivery, and social resources, rather than an isolated psychological endpoint.

### 4.2 Clinical implications

Our findings support several actionable, patient-centred recommendations for DVT follow-up. First, routine screening for anxiety symptoms using the GAD-7 is warranted at diagnosis and at scheduled follow-up visits, with particular attention to younger patients and those with threatened illness perceptions, low perceived social support, or a reported lack of structured counselling at diagnosis. Second, structured use of the counselling checklist at diagnosis—combined with written materials and teach-back to confirm understanding —may reduce uncertainty and fear-inducing miscommunication. Third, patients with clinically significant anxiety symptoms should be offered referral pathways to psychological services (e.g., psychology liaison or community mental health resources), and follow-up protocols should include symptom and functional assessment. Fourth, because role and financial disruption were prominent among younger patients, return-to-work support and involvement of family members should be part of integrated care planning. These recommendations align with the call for value-based, patient-centred VTE care and warrant evaluation in prospective interventional studies.

### 4.3 Limitations

This study has several limitations. The cross-sectional design of the quantitative phase precludes causal inference; although we identified associations among illness perception, social support, and anxiety, the directionality of these relationships remains undetermined. Longitudinal studies are needed to establish whether changes in illness perception over time predict corresponding changes in anxiety trajectories. Second, the study was conducted in only one general hospital in China, which may limit the generalizability of the findings to other healthcare settings or cultural contexts. Third, we did not collect demographic or clinical information on patients who declined to participate or submitted incomplete questionnaires (*n* = 20; effective response rate 89.7%); therefore, a formal comparison between participants and non-participants was not possible, and the potential influence of non-response patterns on the observed prevalence estimate (41.1%) cannot be excluded. Although the high response rate may partly mitigate this concern, the prevalence estimate should be interpreted with this caveat. Fourth, the perceived adequacy of counselling was measured retrospectively using participants’ recollection of information provided at diagnosis. Current anxiety may plausibly influence participants’ recollection or appraisal of counselling received at diagnosis, and social desirability may have affected self-reported responses; differential recall or reverse causality is therefore possible. Fifth, the multivariable model contained seven covariates for 72 anxiety events; although this is within commonly recommended thresholds, the model may be susceptible to overfitting, and formal internal validation was not undertaken. Effect estimates should therefore be interpreted with appropriate caution. Sixth, patients with a documented history of severe psychiatric illness were excluded from the study; this clinically relevant subgroup may have higher psychological burden, and their exclusion may underestimate the overall prevalence of anxiety in the broader LEDVT population and limit generalizability. Seventh, we did not assess anxiety or depression predating the DVT episode; consequently, the study cannot distinguish new-onset, DVT-related anxiety symptoms from pre-existing anxiety vulnerability. Finally, although thematic saturation was achieved in the qualitative sample of 15 participants, the findings may not capture the full spectrum of experiences, particularly among patients who declined to participate in the study.

## 5 Conclusions

In this mixed-methods study, clinically significant anxiety symptoms were common among patients receiving anticoagulant therapy for lower extremity DVT. Younger age, more threatening illness perceptions, inadequate counselling at diagnosis and lower perceived social support were independently associated with anxiety. Qualitative interviews suggested that patients’ anxiety centred on fear of pulmonary embolism, recurrence, post-thrombotic syndrome, anticoagulant-related bleeding, uncertainty about treatment duration, disruption of work and family roles, fragmented information delivery, and insufficient emotional support, with symptom burden and functional limitations featuring prominently in patients’ accounts. Because of the cross-sectional design, modest sample size, and single-centre setting, the findings should be considered exploratory and hypothesis-generating rather than causal or fully generalizable. Nevertheless, the results support incorporating structured counselling, assessment of illness perceptions, symptom and functional assessment, and psychosocial screening into patient-centred DVT follow-up.

## Data Availability

The original contributions presented in the study are included in the article/Supplementary Material, further inquiries can be directed to the corresponding authors.
