# Supplementary material for: Anxiety in patients with lower-extremity deep vein thrombosis: a patient-centred sequential explanatory mixed-methods study of prevalence, associated factors, and patient experiences
Source: Front Med (Lausanne). 2026 Oct 15;13:1933822. doi: 10.3389/fmed.2026.1933822 (PMC13716643; doi:10.3389/fmed.2026.1933822)
Supplement: Supplementary file 1 [file Table1.docx]

Supplementary Materials S2

Maximum-variation sampling matrix for the qualitative phase

The matrix was developed a priori to capture heterogeneity in dimensions considered clinically and psychosocially relevant to the experience of DVT: age group, sex, GAD-7 anxiety severity, DVT extent, presence of concomitant pulmonary embolism, anticoagulant type, time since diagnosis, employment status, and perceived adequacy of counselling at diagnosis. Participants who consented to further contact were listed and screened against the dimensions; individuals were approached in successive waves, prioritizing under-represented sampling cells; after each interview the matrix was updated and the next approach targeted the largest remaining coverage gaps.

| ID | Sex | Age (y) | GAD-7 score | GAD-7 severity | DVT extent | PE | Anticoagulant | Time since diagnosis (mo) | Employment | Counselling adequacy |
| --- | --- | --- | --- | --- | --- | --- | --- | --- | --- | --- |
| P01 | Male | 35 | 18 | Severe | Proximal | No | DOAC | 2 | Employed | Inadequate |
| P02 | Female | 68 | 11 | Moderate | Distal | No | DOAC | 5 | Retired | Adequate |
| P03 | Female | 52 | 10 | Moderate | Proximal | Yes | DOAC | 1 | Sick leave | Inadequate |
| P04 | Male | 70 | 12 | Moderate | Proximal | No | VKA | 6 | Retired | Inadequate |
| P05 | Male | 28 | 16 | Severe | Distal | No | DOAC | 2 | Employed | Adequate |
| P06 | Female | 31 | 19 | Severe | Proximal | No | DOAC | 1.2 | Sick leave | Inadequate |
| P07 | Female | 65 | 13 | Moderate | Distal | No | VKA | 8 | Retired | Inadequate |
| P08 | Male | 47 | 8 | Mild | Proximal | No | DOAC | 3 | Employed | Inadequate |
| P09 | Male | 45 | 14 | Moderate | Proximal | No | DOAC | 4 | Sick leave | Inadequate |
| P10 | Female | 39 | 9 | Mild | Distal | No | DOAC | 7 | Employed | Adequate |
| P11 | Male | 42 | 7 | Mild | Proximal | No | DOAC | 2.5 | Employed | Inadequate |
| P12 | Female | 24 | 15 | Severe | Proximal | No | VKA | 1 | Employed | Inadequate |
| P13 | Male | 56 | 3 | Minimal | Distal | No | DOAC | 10 | Employed | Adequate |
| P14 | Female | 38 | 6 | Mild | Proximal | No | DOAC | 3 | Employed | Adequate |
| P15 | Female | 60 | 2 | Minimal | Distal | No | DOAC | 9 | Retired | Adequate |
