# Supplementary material for: Anxiety in patients with lower-extremity deep vein thrombosis: a patient-centred sequential explanatory mixed-methods study of prevalence, associated factors, and patient experiences
Source: Front Med (Lausanne). 2026 Oct 15;13:1933822. doi: 10.3389/fmed.2026.1933822 (PMC13716643; doi:10.3389/fmed.2026.1933822)
Supplement: Supplementary file 2 [file Table2.docx]

Supplementary Materials S3

Saturation grid for qualitative interviews

| **Interview** | **New codes identified** | **Examples of new codes** | **Cumulative codes** | **New theme** |
| --- | --- | --- | --- | --- |
| P01 | 8 | Fear of PE; activity avoidance; treatment uncertainty | 8 | Yes |
| P02 | 6 | Loss of independence; family role disruption | 14 | Yes |
| P03 | 5 | Need for clinician reassurance; symptom vigilance | 19 | Yes |
| P04 | 5 | Bleeding vigilance; monitoring burden | 24 | Yes |
| P05 | 4 | Internet-related fear; lack of disease understanding | 28 | Yes |
| P06 | 4 | Fear of sudden death; work interruption | 32 | Yes |
| P07 | 3 | Post-thrombotic syndrome concern; mobility restriction | 35 | No |
| P08 | 2 | Financial pressure; medication cost concern | 37 | No |
| P09 | 2 | Uncertain treatment duration; recurrence fear | 39 | No |
| P10 | 1 | Written education needs | 40 | No |
| P11 | 1 | Body monitoring and repeated checking | 41 | No |
| P12 | 0 | None | 41 | No |
| P13 | 0 | None | 41 | No |
| P14 | 0 | None | 41 | No |
| P15 | 0 | None | 41 | No |

Meaning saturation was defined a priori as the point at which no new codes or substantially new interpretations relevant to the study aims emerged in three consecutive interviews. No new codes were identified from the 12th interview onward; three additional interviews (P13–P15) were conducted to confirm informational redundancy.
