# Supplementary material for: Anxiety in patients with lower-extremity deep vein thrombosis: a patient-centred sequential explanatory mixed-methods study of prevalence, associated factors, and patient experiences
Source: Front Med (Lausanne). 2026 Oct 15;13:1933822. doi: 10.3389/fmed.2026.1933822 (PMC13716643; doi:10.3389/fmed.2026.1933822)
Supplement: Supplementary file 3 [file Table3.docx]

Supplementary Materials S4

Semi-structured interview guide

The guide was developed on the basis of the quantitative findings and the existing VTE patient-outcome literature, and was refined through pilot interviews with two patients with DVT. The seven core domains covered are listed below with example probes.

| Domain | Example probes |
| --- | --- |
| 1. Personal experiences of being diagnosed with and living with DVT | Can you tell me about the time you were diagnosed? What has it been like living with DVT since then? |
| 2. Perceptions of pulmonary embolism, recurrence, bleeding, post-thrombotic syndrome, and prognosis | What do you understand about the risks of this condition? How worried are you about these risks? |
| 3. Emotional responses to anticoagulation management | How do you feel about taking anticoagulant medication? What concerns, if any, do you have? |
| 4. Impact on daily activities, family roles, work, and finances | How has DVT affected your daily life, your work, and your family responsibilities? |
| 5. Information received at diagnosis and perceptions of clinician communication | What information were you given at diagnosis? How did the doctor explain things to you? |
| 6. Sources of social and professional support | Who supports you in managing your condition? What kind of support is most helpful? |
| 7. Unmet supportive care needs | What additional information, help, or support would you have wanted? |

Pilot testing and refinement. The guide was pilot-tested with two patients with DVT before formal data collection. Pilot testing led to the following refinements: (1) probes on pulmonary embolism and recurrence risk were reworded in simpler, lay language to reduce misunderstanding; (2) probes on the impact of DVT on work, finances, and family roles were reordered and grouped earlier in the guide, as pilot participants identified these as the most emotionally salient concerns.
