# Supplementary material for: Anxiety in patients with lower-extremity deep vein thrombosis: a patient-centred sequential explanatory mixed-methods study of prevalence, associated factors, and patient experiences
Source: Front Med (Lausanne). 2026 Oct 15;13:1933822. doi: 10.3389/fmed.2026.1933822 (PMC13716643; doi:10.3389/fmed.2026.1933822)
Supplement: Supplementary file 4 [file Table4.docx]

**Supplementary Materials S1**

**Eight-item Structured Counselling Checklist**

Administration: the checklist was completed by the research team on the basis of participants’ recollection of the information they received at the time of DVT diagnosis. Each item was scored 0 (no) or 1 (yes). The total score ranged from 0 to 8. Adequate counselling was defined a priori as a score ≥ 6.

| Item | Content (recalled by participant) |
| --- | --- |
| 1 | The nature and causes of DVT were explained |
| 2 | Pulmonary embolism risk and warning signs were explained |
| 3 | Recurrence risk and prevention were explained |
| 4 | Bleeding risk and management were explained |
| 5 | Anticoagulation duration and follow-up plan were explained |
| 6 | Post-thrombotic syndrome and the expected symptom trajectory were explained |
| 7 | Written or electronic educational materials were provided |
| 8 | The participant had the opportunity to ask questions, with confirmation of understanding |

Scoring and cut-off justification: a threshold of ≥ 6 (of 8) was defined a priori because it requires recall of the core safety-critical components of diagnosis counselling — pulmonary embolism warning signs, bleeding management, recurrence prevention, and treatment/follow-up planning (items 2, 4, 3, and 5, respectively, at least three of which must be affirmed together with any combination of the remaining items to reach 6).

Instrument development: the checklist was developed for this study on the basis of VTE patient-education content recommended in clinical practice guidelines and input from a multidisciplinary expert panel comprising three vascular surgeons, two specialist nurses, and one clinical psychologist. It was refined through pilot testing in 10 patients with DVT. Because the eight items assess objectively recallable components of diagnosis counselling rather than a unidimensional attitudinal scale, internal consistency (Cronbach’s α) was not calculated.
